# A global-scale multidecadal variability driven by Atlantic multidecadal oscillation

**DOI:** 10.1093/nsr/nwz216

**Published:** 2019-12-24

**Authors:** Young-Min Yang, Soon-Il An, Bin Wang, Jae Heung Park

**Affiliations:** Department of Atmospheric Science, Key Laboratory of Meteorological Disaster of Ministry of Education, Joint International Research Laboratory of Climate and Environment Change, Collaborative Innovation Center on Forecast and Evaluation of Meteorological Disasters and Earth System Modeling Center, Nanjing University of Information Science and Technology, Nanjing 210044, China; Department of Atmospheric Sciences and International Pacific Research Center, University of Hawaii, Honolulu 96822, USA; Department of Atmospheric Sciences and Irreversible Climate Change Research Center, Yonsei University, Seoul 03722, Korea; Department of Atmospheric Science, Key Laboratory of Meteorological Disaster of Ministry of Education, Joint International Research Laboratory of Climate and Environment Change, Collaborative Innovation Center on Forecast and Evaluation of Meteorological Disasters and Earth System Modeling Center, Nanjing University of Information Science and Technology, Nanjing 210044, China; Department of Atmospheric Sciences and International Pacific Research Center, University of Hawaii, Honolulu 96822, USA; Division of Environmental Science and Engineering, Pohang University of Science and Technology, Pohang 37673, Korea

**Keywords:** global-scale multidecadal variability, Atlantic multidecadal oscillation, atmospheric teleconnection, interdecadal Pacific oscillation

## Abstract

Observational analysis shows that there is a predominant global-scale multidecadal variability (GMV) of sea-surface temperature (SST). Its horizontal pattern resembles that of the interdecadal Pacific oscillation (IPO) in the Pacific and the Atlantic multidecadal oscillation (AMO) in the Atlantic Ocean, which could affect global precipitation and temperature over the globe. Here, we demonstrate that the GMV could be driven by the AMO through atmospheric teleconnections and atmosphere–ocean coupling processes. Observations reveal a strong negative correlation when AMO leads GMV by approximately 4–8 years. Pacemaker experiments using a climate model driven by observed AMO signals reveal that the tropical Atlantic warm SST anomalies of AMO initiate anomalous cooling in the equatorial central-eastern Pacific through atmospheric teleconnections. Anticyclonic anomalies in the North and South Pacific induce equatorward winds along the coasts of North and South America, contributing to further cooling. The upper-ocean dynamics plays a minor role in GMV formation but contributes to a delayed response of the IPO to the AMO forcing. The possible impact of the GMV on AMO was also tested by prescribing only Pacific SST in the model; however, the model could not reproduce the observed phase relationship between the AMO and the GMV. These results support the hypothesis that the Atlantic Ocean plays a key role in the multidecadal variability of global SST.

## Teaser

Long-term variability of the North Atlantic ocean generates a global SST variability with multi-decadal time scale through atmospheric teleconnection.

## INTRODUCTION

Global sea-surface temperature (SST) shows two dominant decadal variabilities: interdecadal Pacific oscillation (IPO) and Atlantic multidecadal oscillation (AMO), which largely affect global climate change. The IPO exhibits a 40- to 60-year oscillation covering the entire Pacific basin [1–4]. If the IPO resembles the El Niño-Southern oscillation (ENSO) with a wider meridional extension over the tropical Pacific, then its SST anomaly pattern resembles that of the Pacific decadal oscillation (PDO) over the northern mid-latitude West and Central Pacific. Here, the PDO might be separable from the IPO according to the target area and predominant timescale [1–3,5–8]. The IPO affects precipitation over eastern Australia, southern Africa, western Canada, India, East Asia and the Southwest USA [1–3,9,10]. It can also cause severe droughts throughout India and the continental USA [5]. Furthermore, it modifies ENSO impacts over Australia and the East Pacific, and influences the South Pacific convergence zone near Australia [11,12], prevailing track of tropical cyclones [13] and the ENSO–East Asian summer-monsoon relationship [14–17]. Recent global-warming deceleration was also claimed to be related to the IPO [18,19].

Several hypotheses on the origins of IPO have been proposed including: low-frequency variation through a reddening process caused by stochastic atmospheric forces [3,20]; SST changes through dynamical advection associated with North Pacific gyre oscillation [21] or upper-ocean circulation [22]; and SST anomalies initiated by Kuroshio currents reinforced by unstable air–sea interactions in the mid-latitudes [23–26]. Recently, mechanisms based on wind-driven upper-ocean circulation have also been proposed, indicating that easterly wind anomalies decrease Central and East Pacific SST and increase West Pacific SST by enhancing upwelling and deepening thermocline, respectively. Based on this theory, the period of the IPO depends on the oceanic Rossby wave-propagation speed [3,19] and tropical forcing can lead to extra-tropical atmospheric responses by the ocean dynamics with a delayed time [5,27]. However, the dynamical and physical processes behind IPO are not well understood. Regarding the source of the easterly winds or atmospheric forces driving upper-ocean circulation, previous studies mentioned that a warm phase of the AMO could enhance the mean easterly and modify ENSO variability in the East Pacific [28,29]. A suite of numerical experiments showed that AMO can induce the IPO pattern (e.g. Pacific decadal oscillation-like anomalies in the north Pacific and symmetric SST anomalies in the south Pacific [30]).

## RESULTS

### The GMV connected with AMO

**Figure 1.**
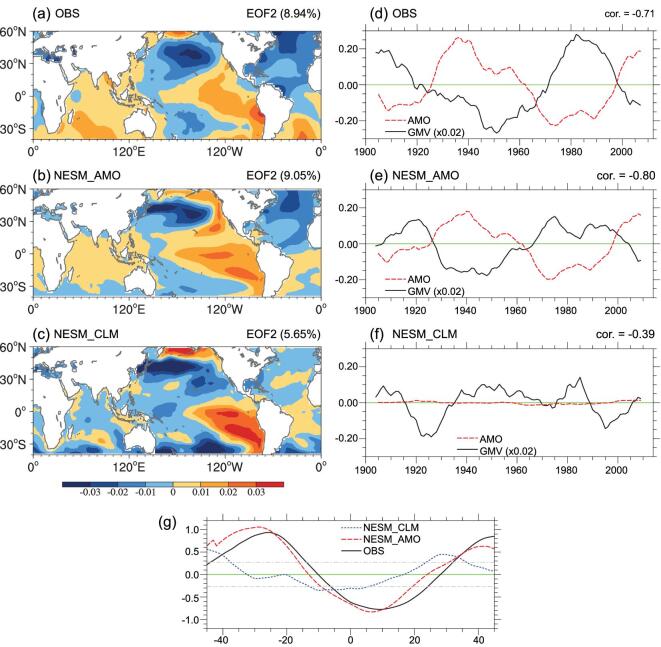
(a–c) Horizontal patterns of GMV from observation and model simulations with observed SST and climatological SST over the AMO region (0°–70°N, 80°W–0°) on decadal timescales (11-year running average, 1900–2014). (d–f) The corresponding time series of GMV (black line) and AMO (red line) from observation and model simulations. GMV is defined as the second EOF mode of global SST (0°–360°E, 40°S–60°N). AMO is defined as SST averaged over 300°–360°E and 0°–70°N. Long-term linear trends in the SST data were removed prior to regression analysis. Additionally, the first and last 11 years were excluded from analysis. (g) Lead–lag phase relationship between GMV and AMO time series from observation data and model simulations. Dashed gray horizontal lines represent 95% significance levels. The lag is positive (negative) when the AMO leads (lags).

Using observed analysis, our study suggests that a global SST shows a dominant variability with multidecadal time scales (hereafter GMV). Figure 1a presents the observed positive GMV pattern and corresponding time series from 1900 to 2014. Here, GMV is defined as the second empirical orthogonal function (EOF) mode of global SST (see the ‘Methods’ section). Note that the first EOF of global SST represents the global-warming trends (see Supplementary Fig. 1). The horizontal pattern of the GMV is characterized by three triangular shapes. The first is an ENSO-like pattern in the tropical Pacific. The second is a PDO-like pattern in the North Pacific. The third lies in the South Pacific and has a relatively weak magnitude. In the North Atlantic, negative SST anomalies can be observed with a horizontal pattern similar to an AMO negative phase. The principal component (PC) time series reveals that the GMV has two positive phases (1900–21, 1972–98) and two negative phases (1922–72, 1999–2014) (Fig. 1d, black line). A comparison of the time series of GMV and AMO (Fig. 1d, red line) reveals an overall out-of-phase relationship. However, the phase change in AMO tends to occur a few years ahead of GMV. The lead–lag correlation between the AMO and GMV time series (Fig. 1g and Supplementary Fig. 5) indicates that positive AMO leads negative GMV by 4–8 years. The interval from the maximum negative lagged correlation to next maximum positive correlation is approximately 30–36 years, which is somewhat consistent with the half-period of GMV shown in the time series in Fig. 1d, implying a possible role for the AMO as a pacemaker for GMV. Apart from the role of AMO, the tropical Pacific was claimed as a pacemaker of the global-warming staircase through intermittently inducing the La Niña-like cooling with a multidecadal timescale. Therefore, a failure in the climate-model simulations of the observed early twenty-first-century warming slowdown is attributable to the prediction failure of the amplitude and timing of IPO [31–35]. However, the pacemaker of IPO has still not been clearly identified.

To explore a possible connection between tropical Pacific climate and AMO, we analysed observed

data and conducted a series of AMO and GMV pacemaker experiments using a fully coupled climate model to identify the relationship between AMO and GMV, and analyse how AMO contributes to the generation of interdecadal variability over the Pacific. Specifically, the tropical upper-tropospheric bridge mechanism between the Pacific and Atlantic Oceans and the role of upper-ocean circulation in GMV were investigated. The results presented in this paper will elucidate the interplay between AMO and GMV, and how AMO drives GMV.

**Figure 2.**
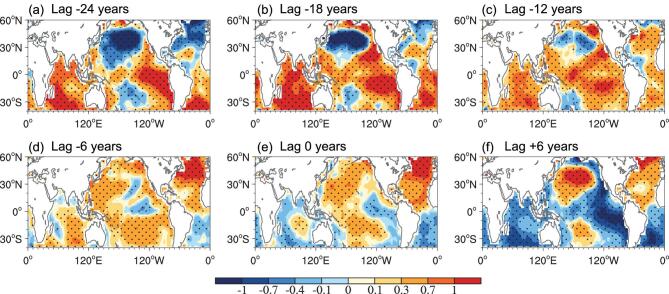
Horizontal structure of global SST anomalies in observation data depicted by regressing SST anomalies (K) onto the AMO index (0°–70°N, 80°W–0°) with (a) lag –24 years, (b) lag –18 years, (c) lag –12 years, (d) lag –6 years, (e) lag 0 years and (f) lag +6 years on decadal timescales (11-year running average, 1900–2018). Long-term linear trends in the SST data were removed prior to regression analysis and the first and last 11 years were excluded from analysis. The dotted area represents a 95% significance level.

To analyse the AMO–GMV relationship further, we compared regressed global SST anomalies to the AMO index with different lead times (–24 to 6 years) using observations from 1900 to 2014 (Fig. 2). In the decaying negative AMO phase (lead time of –24 years), the horizontal pattern of global SST anomalies resembles a positive peak phase of GMV and its pattern correlation with the data in Fig. 1a is 0.87. When the negative AMO phase transitions into a positive phase (lead time of –18 to –12 years), the cold anomalies in the North and South Pacific gradually decay, but the warming in the tropical Pacific is maintained with reduced magnitude. At a lead time of –6 years, the North Atlantic warms up significantly and a positive AMO phase is established. Additionally, the original negative SST anomalies disappear, except for minor cooling in the Northeast Pacific. Notably, a cooling pattern emerges over the NINO3.4 region (5°S–5°N, 120°–170°W). When a positive AMO phase reaches a peak (lead time of 0 years), the negative SST anomalies in the East Pacific extend eastward, then poleward along the coasts of South and North America. Six years after peak positive AMO, cold SST anomalies develop in the East Pacific triangular region and positive SST anomalies are strengthened in the North and South Pacific, which is very similar to a negative peak GMV phase (pattern correlation coefficient of –0.91). The subsequent evolution of SST anomalies is almost a mirror image of the previous half-cycle (Supplementary Fig. 3). These results strongly suggest that North Atlantic and Pacific SST are highly correlated on a multidecadal timescale and that the lead–lag relationship is robust.

To explore whether GMV may be driven by AMO, we conducted two types of numerical experiments using an earth-system model (see the ‘Methods’ section). In the first experiment, observed AMO signals were engaged in the model by nudging the observed SST anomalies from 1900 to 2014 only over the AMO region (80°W–0°, 0°–70°N) (NESM_AMO). In the second experiment, AMO signals were intentionally suppressed by nudging SST based on climatology in the same region (NESM_CLIM). In all other regions, the model was freely coupled. As shown in Fig. 1b, NESM_AMO reproduced the observed GMV pattern and its corresponding time variation reasonably realistically. The percentage of variance (9.05%) is similar to the observed value (8.95%). The pattern correlation of GMV between NESM_AMO (Fig. 1b) and observations (Fig. 1a) is 0.68 and the temporal correlation between the PC time series is 0.76 (Supplementary Fig. 4). Both correlations are statistically significant with a confidence level of 95%. Just as in the observations, the GMV index from NESM_AMO has two positive (1900–26, 1967–2003) and two negative (1927–66, 2004–14) phases. Furthermore, the amplitude of the GMV index is close to the observed value. Additionally, as shown in Fig. 1g (red line), the lead–lag phase relationship between the GMV and AMO indices obtained from NESM_AMO is consistent with observations. The maximum negative correlation when AMO leads GMV is 0.82, which is similar to the observed value of 0.79. However, NESM_CLIM does not reproduce the GMV pattern accurately, exhibiting a relatively low pattern correlation with the observations (0.46) (Fig. 1c). Although the anomalous SST patterns have similarities overall, the locations and intensities of the anomaly centers are different. NESM_CLIM also failed to simulate the temporal characteristics of the GMV index in terms of amplitude and phase (Fig. 1f). Therefore, these experiments strongly support the idea that SST associations with AMO are crucial for generating GMV patterns and tracking their temporal evolution.

### How does AMO drive GMV?

**Figure 3.**
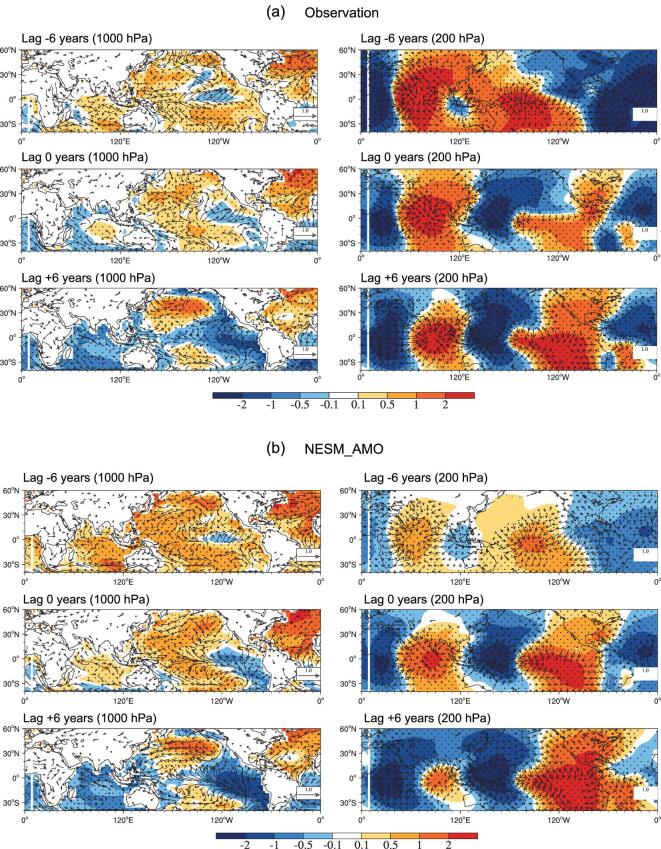
Horizontal structure of global SST (shading, K) and surface wind vectors (m s^–1^) (left panel), and 200-hPa velocity potential (shading, 10^5^ m^2^ s^−1^) and divergent winds (m s^−1^) (right panel) from (a) observation and (b) NESM_AMO, depicted by the regressing SST (K), surface wind vectors (m s^−1^), 200-hPa velocity potential (10^5^ m^2^ s^−1^) and divergent winds (m s^−1^) onto the AMO index (0°–70°N, 80°W–0°) with lead times of –6 years (top), 0 years (middle) and 6 years (bottom). Eleven years of running average data were used and long-term linear trends in the SST data were removed prior to regression analysis. The dotted area represents a 95% significance level.

To investigate a possible mechanism for AMO driving GMV patterns, we examined the horizontal atmospheric circulation associated with AMO through observation and NESM_AMO simulation. Figure 3a presents the observed horizontal circulation at the surface (1000 hPa) and upper troposphere (200 hPa) in relation to the AMO index with lead times ranging from –6 to 6 years. For a lag of –6 years, the warm SST anomalies associated with AMO generate ascending motions with strong upper-level divergent flows that are connected to upper-level convergences in the central subtropical North and South Pacific, as well as the equatorial central Pacific, where the descending motions prevail (Fig. 3a, upper panel). The corresponding low-level pressure values contain two anomalous high values in the central North and South Pacific (Fig. 3a, upper panel). The descending motions in the central Pacific produce surface easterly anomalies over the central-to-western Pacific, which intensifies the equatorial thermocline slope. At the same time, the anomalous ascending branch of Walker circulation over the equatorial western Pacific gradually builds up with the aid of the warmer SST anomalies and the low-level convergence (Fig. 3a, upper panel). These two effects could reinforce the cooling of the cold-tongue SST anomalies. With lags of –6 to 0 years, anticyclonic anomalies in the North Pacific may enhance the northerlies along the west coast of North America and the trade winds of the East Pacific. The enhanced alongshore northerly reinforces coastal upwelling and the offshore Ekman transport of cold water, and enhanced trade winds boost evaporative cooling (Supplementary Fig. 6). As a result, both factors tend to cool the northeast tropical Pacific (Fig. 3b, middle panel). Similarly, the anticyclonic anomaly in the Southeast Pacific also enhances cooling. As a result, central equatorial Pacific cooling develops into large-scale cooling in the triangular region of the tropical East Pacific with a lag of 6 years (Fig. 3c, middle panel). Meanwhile, the ascending motion in the far West Pacific and descending motion in the central Pacific enhance surface anomalous easterlies, extending East Pacific cooling westward along the equator. The resulting enhanced zonal contrast of equatorial Pacific SST reinforces the trade winds by enhancing pressure gradients. This air–sea coupled feedback, which is known as Bjerknes feedback [36], can amplify SST anomalies. These results suggest that tropical easterly anomalies and anticyclonic anomalies excited by AMO in the North and South Pacific contribute to the development of the negative phase of GMV in the East Pacific. This also suggests that GMV may be intensified by positive air–sea coupled feedback after being triggered by AMO.

The NESM_AMO simulation (Fig. 3b) captures the observed horizontal patterns of both SST and atmospheric circulation anomalies well, as seen in the significant pattern correlations of about 0.68–0.73 for a lag of –6 to 6 years, while the magnitude is relatively stronger in the model. For the lags of –36 to –24 years, the sequential processes on how the negative AMO index drives GMV is similar to that which appears for the lags of –6 to 6 years.

**Figure 4.**
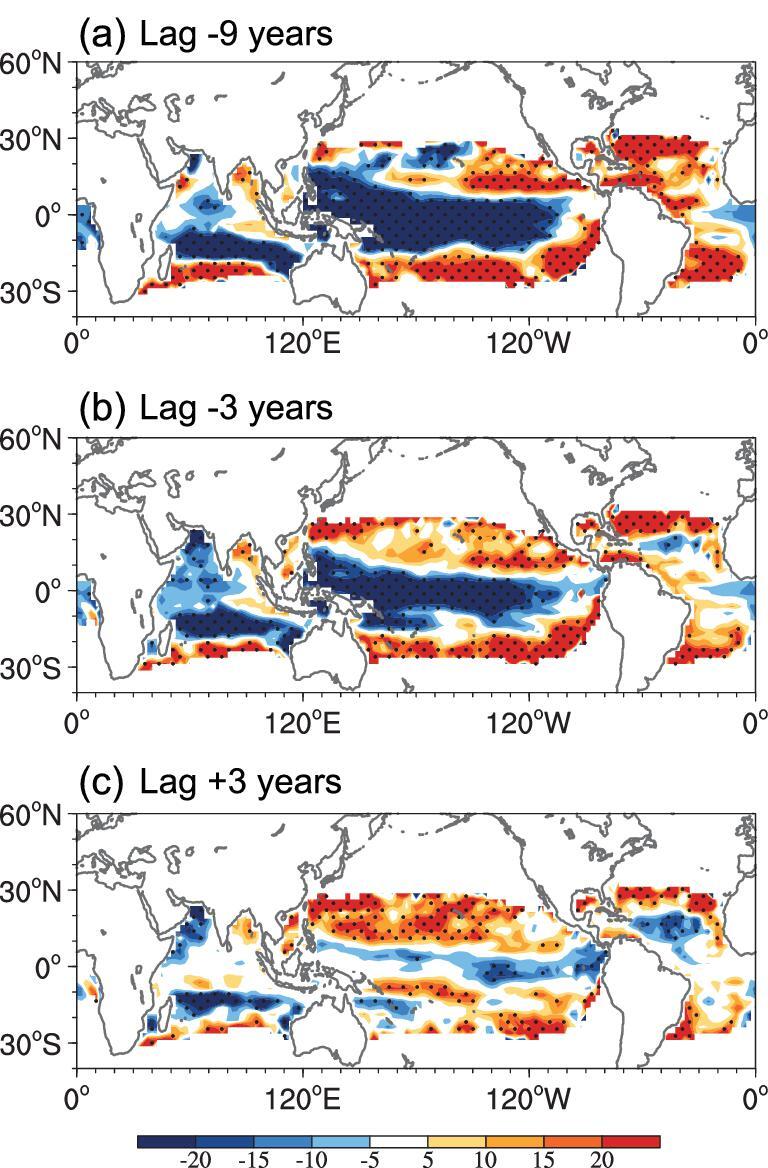
Horizontal structure of thermocline depth (m^−1^) at 20°C from NESM_AMO depicted by regressing thermocline depth onto the AMO index (0°–70°N, 80°W–0°) with lead times of (a) –9 years, (b) –3 years and (c) 3 years. Eleven years of running average data were used and long-term linear trends in the SST data were removed prior to regression analysis. The dotted area represents a 95% significance level.

Ocean memory measured based on upper-ocean heat content is known to be a driver of a long-term variability and can cause interdecadal variability in an ocean. To investigate the potential role of internal ocean dynamics on the evolution of GMV, we examined the relationship between thermocline depth and the AMO index using NESM_AMO, as shown in Fig. 4. With a lag of –9 years relative to positive AMO, the thermocline depth is shallow in the West Pacific and relatively deep in the East Pacific. Six years later (lag of –3 years), the thermocline becomes deeper in the West Pacific and relatively shallow in the far East Pacific, which is likely caused by the enhanced trade winds associated with positive AMO. These factors suggest that changes in thermocline depth anomalies lead to the initial development of GMV in the equatorial East Pacific. It is noteworthy that GMV responses are delayed with respect to AMO forces. In general, SST may fluctuate within 2–3 months as a response to surface heat flux over a much longer period based on the ocean dynamical processes related to atmospheric forces. As shown in Fig. 1, the observed GMV lags behind AMO by approximately 4–6 years. This delayed response is related to slow ocean dynamical adjustments in the tropical Pacific basin and AMO-induced atmospheric teleconnections. It has a delayed impact on SST based on ocean dynamical thermal advection.

## DISCUSSION

Observations show that GMV and AMO are significantly correlated with a positive AMO phase leading a negative GMV phase by 4–8 years. Climate-model simulation based on observed SST over AMO regions shows that atmospheric teleconnections between the tropical Atlantic and East Pacific are a key factor linking AMO and GMV. The tropical Atlantic warm SST anomalies associated with positive AMO generate surface wind anomalies in the central and East Pacific, which initiate a negative GMV phase in the equatorial central and eastern tropical Pacific. Cold SST anomalies are intensified in the East Pacific triangular region by enhanced wind–evaporation–SST feedback during developing AMO. In contrast, the delayed response of SST to AMO forces in the Pacific is largely caused by delayed thermocline adjustment based on SST and wind anomalies associated with the atmospheric responses of remote AMO forces.

We also explore a possible role of external forcing (e.g. volcanic and aerosol emissions) on the occurrence of GMV by conducting an AMO pacemaker experiment with the pre-industrial (1850s) external forcing (NESM_PI). As shown in Supplementary Fig. 8, the horizontal pattern of GMV is similar to those of NESM_AMO but the corresponding amplitude is weaker. The out-of-phase relationship between GMV (EOF2 PC) and AMO index in NESM_PI is also visible but the maximum peaks are slightly weaker. The Power spectrum analysis shows that GMV from NEM_PI has a dominant multidecadal timescale of 60 years. On the whole, the role of the volcanic and aerosol effects on GMV seems to be minor.

Finally, because positive GMV leads negative AMO by 25–30 years (Fig. 1g), we tested the possibility that GMV could impact AMO. To explore this concept, we conducted an GMV pacemaker experiment (NESM_IPO) using the same model from our previous experiments, where observed SSTs in the Pacific region (120°–280°E, 60S°–70°N) were collected from 1900 to 2014 (see the ‘Methods’ section). As shown in Supplementary Fig. 9, the horizontal pattern of AMO was not accurately reproduced by the NESM_IPO experiment. Warm SST anomalies only occur over a range of 20°–40°N, whereas cold SST anomalies occur in the tropical and high-latitude Atlantic Ocean. These results clearly differ from the observations (e.g. Fig. 1a). The period of simulated AMO (10–40 years) is shorter than the observed period (40–80 years) and its magnitude is much smaller than the observed magnitude. These results indicate that GMV cannot generate AMO.

Our model simulations were conducted using a single model. Similar studies using different climate models are required to examine the degree of model dependency of the results. Regardless, strong similarities between observations and model simulations strongly support our hypothesis regarding the role of AMO in driving GMV. It would be worthwhile to examine the relationship between the multidecadal variability of the Indian Ocean and AMO in a future project.

## METHODS

### Earth-system model

We used the third version of the Nanjing University of Information Science and Technology Earth System Model (NESM3.0) [37–40]. NESM3.0 consists of atmosphere, ocean, sea ice and land models, which are fully coupled by an explicit coupler. The resolution of the atmosphere model is T63L47. The ocean model has a grid resolution of 1° with the meridional resolution refined to 1/3° over the equatorial region. It uses 46 vertical layers, with the first 15 layers in the top 100 m. NESM3.0 simulates not only reasonable climatology, but also key characteristics of PDO [23,24].

### Design of numerical experiments

Two experiments were conducted using the coupled model discussed above to examine the impact of AMO on GMV. The first model simulation was run using the observed SST anomalies (removing linear long-term trends, NESM_AMO) and the second was run using the climatological SST (AMO_CLIM) specified over the AMO domain (0°–70°N, 80W°–0°) with a 1-day nudging timescale. Additionally, a model simulation with the observed SST specified over the Pacific (120°–280°E, 70°S–70°N) was conducted to examine the possible effects of GMV on AMO. There was no further tuning of the parameters in the simulations and the model components were freely coupled with each other over the globe, except for the region where the SST was specified. For all experiments, the model was integrated from the years of 1870–2014 using external forces (greenhouse gases, solar constant, aerosol concentration, ozone, etc.) based on coupled model intercomparison project phase six (CMIP6) protocols and data from the past 115 years (1900–2014). The initial conditions for integration were obtained from a historical run based on the CMIP6 protocols.

### Observed data and diagnostic methods

For the monthly mean SST, we used the National Oceanic and Atmospheric Administration Extended Reconstructed SST version 4 [41]. The ocean temperature and salinity derived from the European Center for Medium-Range Weather Forecasts ocean reanalysis and ocean heat content data sets were used as observation data [42]. GMV was defined by the second EOF pattern and its PC of the global SST, which were smoothed using an 11-year running average. AMO was defined as the averaged SST anomalies over 0°–60°N and 300°–360°E with an 11-year running average [1,2]. Wind data were extracted from a merged National Centers for Environmental Prediction data set from NOAA-CIRES Twentieth Century Reanalysis [43] (1871–1978) and NCEP/DOE Reanalysis 2 (1979–2018).

### Data and materials availability

All observed data used in this study are publicly available and newly generated data from this study are available upon request.

## Supplementary Material

nwz216_Supplemental_File
