# Supplementary material for: A global-scale multidecadal variability driven by Atlantic multidecadal oscillation
Source: Natl Sci Rev. 2019 Dec 24;7(7):1190–7. doi: 10.1093/nsr/nwz216 (PMC8288868; doi:10.1093/nsr/nwz216)
Supplement: nwz216_Supplemental_File [file nwz216_supplemental_file.docx]

**Supporting Online Material for:**

**A Global-scale Multidecadal variability Driven by the Atlantic Multidecadal Oscillation**

Young-Min Yang^1,2^, Soon-Il An ^3*^ Jae Heung Park^2^ and Bin Wang ^1,2*^

^1^ Key Laboratory of Meteorological Disaster of Ministry of Education and Earth System Modeling Center, Nanjing University of Information Science and Technology, Nanjing, China

^2^ Department of Atmospheric Sciences and International Pacific Research Center, University of Hawaii, Honolulu, Hawaii 96822, USA

^3^ Department of Atmospheric Sciences and Irreversible Climate Change Research Center, Yonsei University, Seoul 03722, Korea

**
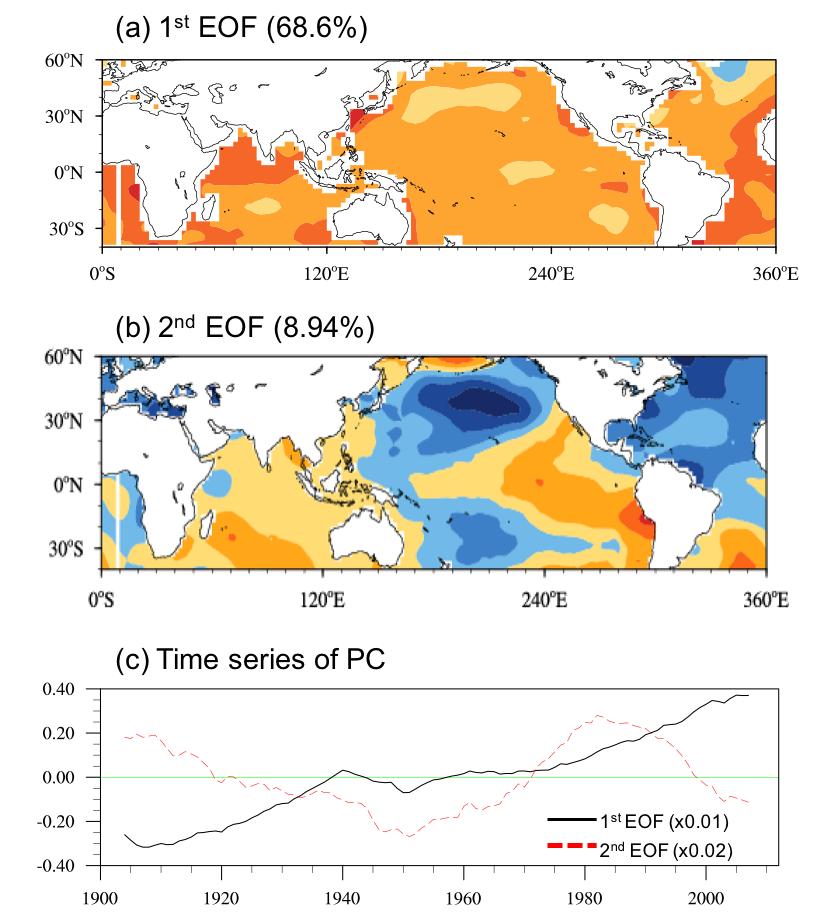
**

**Figure S1.** (a-b) Horizontal pattern and (c) time series of GMV from observation. GMV is defined as the second EOF modes of global SST (0°-360°E, 40°S-60°N). The long-term linear trends in SST data were removed prior to the regression analysis and first and last 11 yrs are excluded for analysis.

**
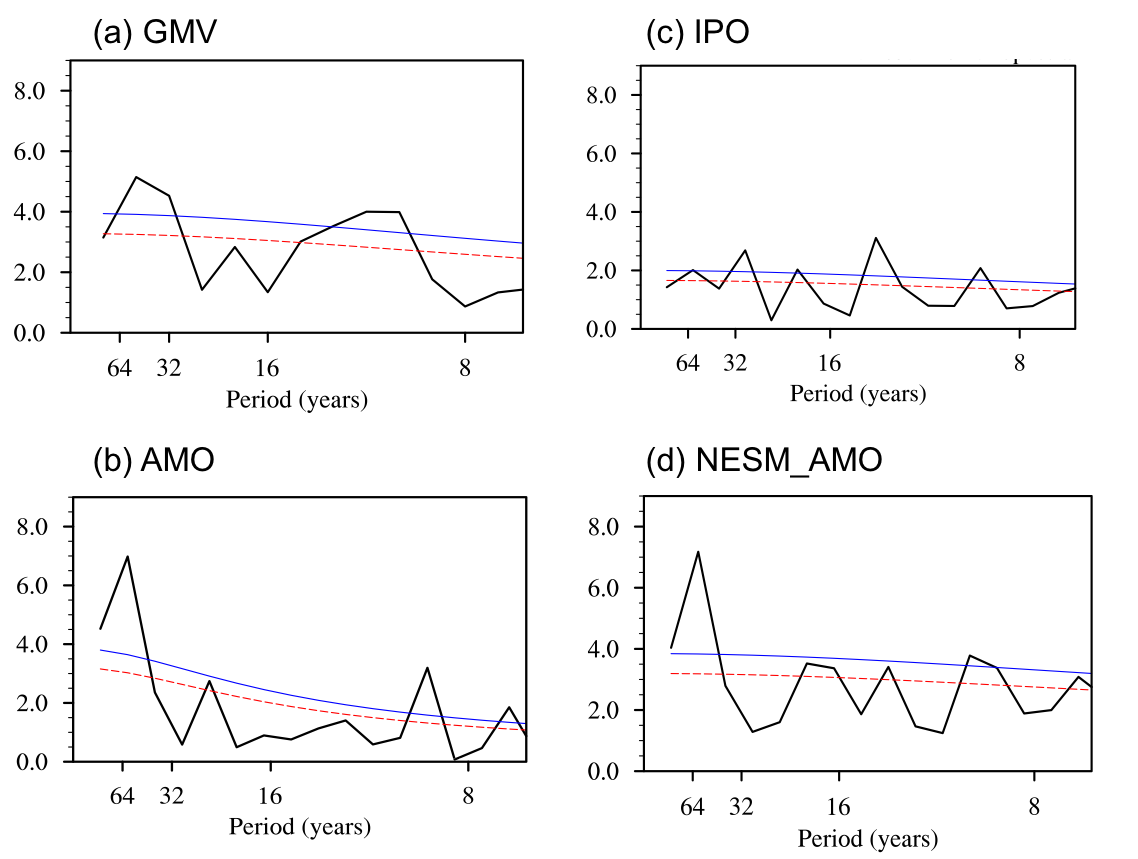
**

**Figure S2.** Power spectral analysis of (a-c) the GMV, IPO, AMO from observation and (d) GMV from NESM_AMO simulation. The long-term trends in AMO index are removed before spectral analysis. Annual mean data are used for all indices. The red dash lines and blue lines indicate the corresponding red noise spectra and 95% confidence levels for power spectra, respectively.

**
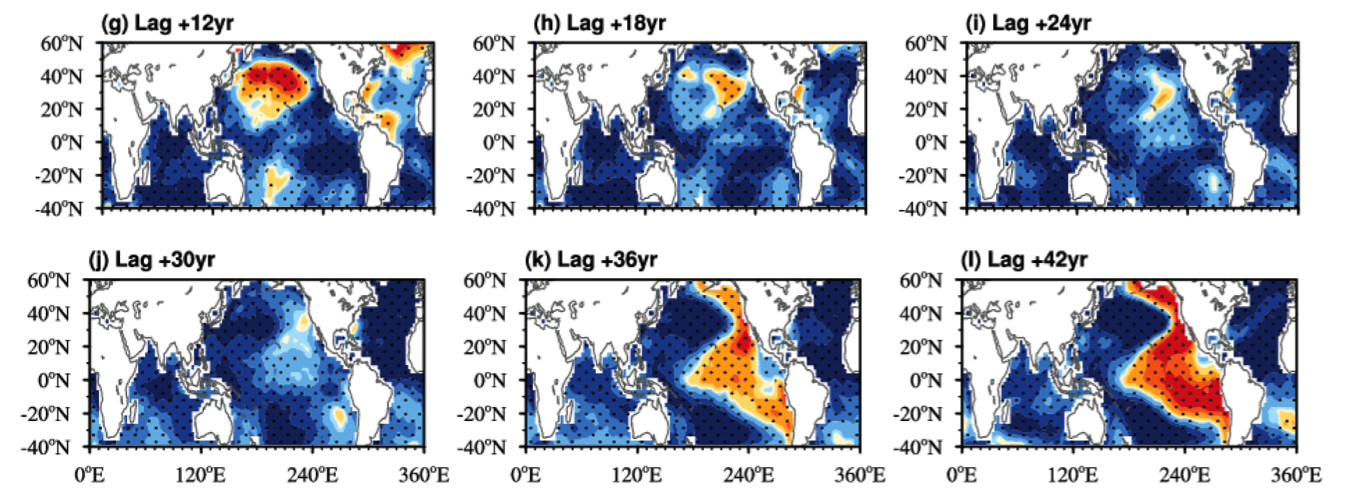
**

**Figure S3.** Horizontal structure of global SST in observation, depicted by the regressed SST (K) onto the AMO index (0°S–70°N, 80°W–0°W) with different lead times at decadal timescales (11 yrs running mean, 1920–2018). The long-term linear trends in SST data were removed prior to the regression analysis.


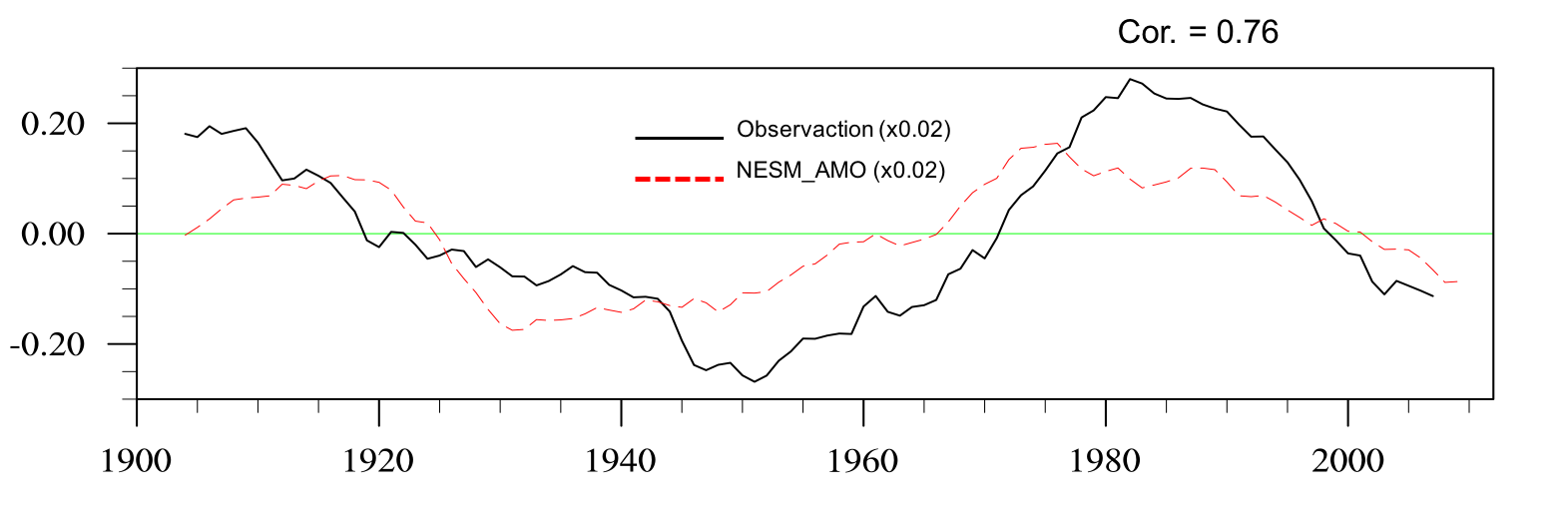


**Figure S4.** Time series of the GMV from observation and NESM_AMO.


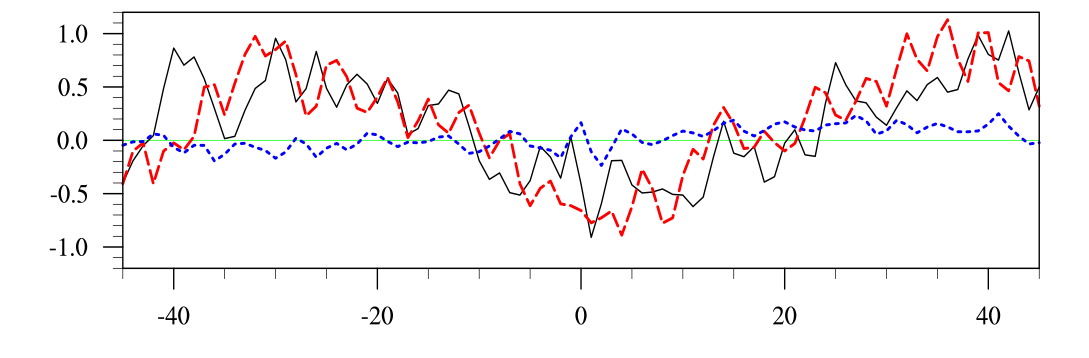


**Figure S5.** Same as Figure 1g but for annual mean data.


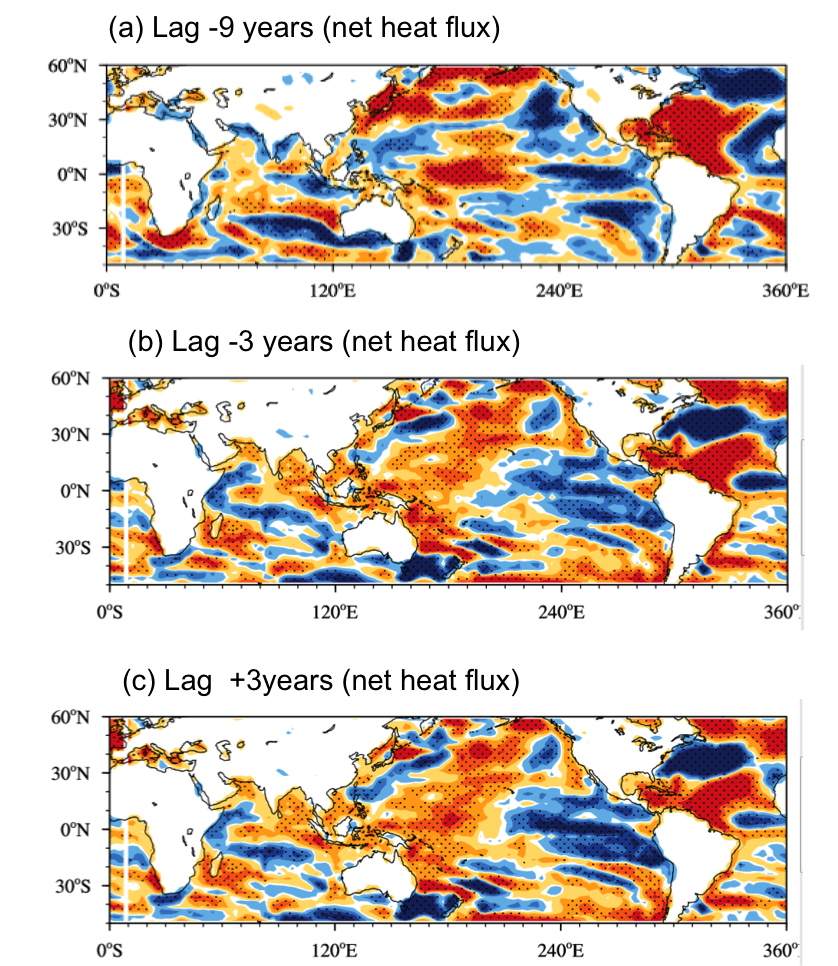


**Figure S6.** The lagged regression coefficients of net surface heat flux (W m^-2^) anomalies on the AMO index with a lag of (a) -9 years, (b) -3 years, and (c) +3 years from NESM_AMO. The latitude-depth structure of ocean circulation averaged over 200°E–260°E is depicted by the regressed ocean circulation onto the AMO index (0°S–70°N, 80°W–0°W) with lead times of -9 years. Eleven years of running mean data are used and long-term linear trends in SST data were removed prior to the regression analysis.


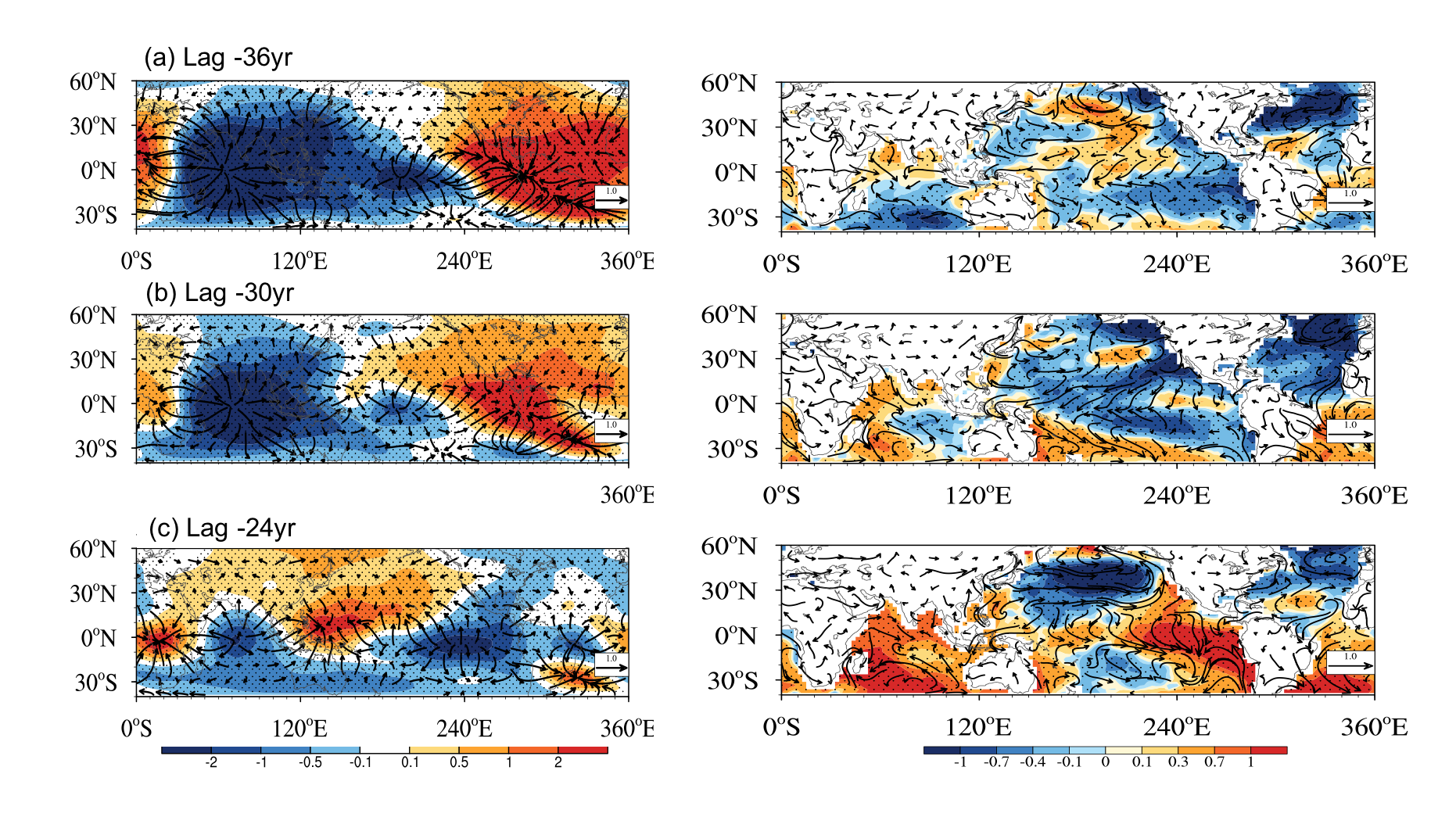


**Figure S7**. Horizontal structure of (a-c) global SST (shading, K) and surface wind vectors (m s^-1^), and (d-e) 200 hPa velocity potential (shading, 10^5^ m^2^ s^-1^) and divergent winds (m s^-1^) from NESM_AMO, depicted by the regressed SST (K), surface wind vectors (m s^-1^), 200 hPa velocity potential (10^5^ m^2^ s^-1^) and divergent winds (m s^-1^) onto the AMO index (0°S–70°N, 80W°–0°W) with lead times of -36 years (top), -30 years (middle) and -24 years (bottom). 11yrs running mean data are used and long-term linear trends in SST data were removed prior to the regression analysis. The dotted area represents 95% significance level.

**
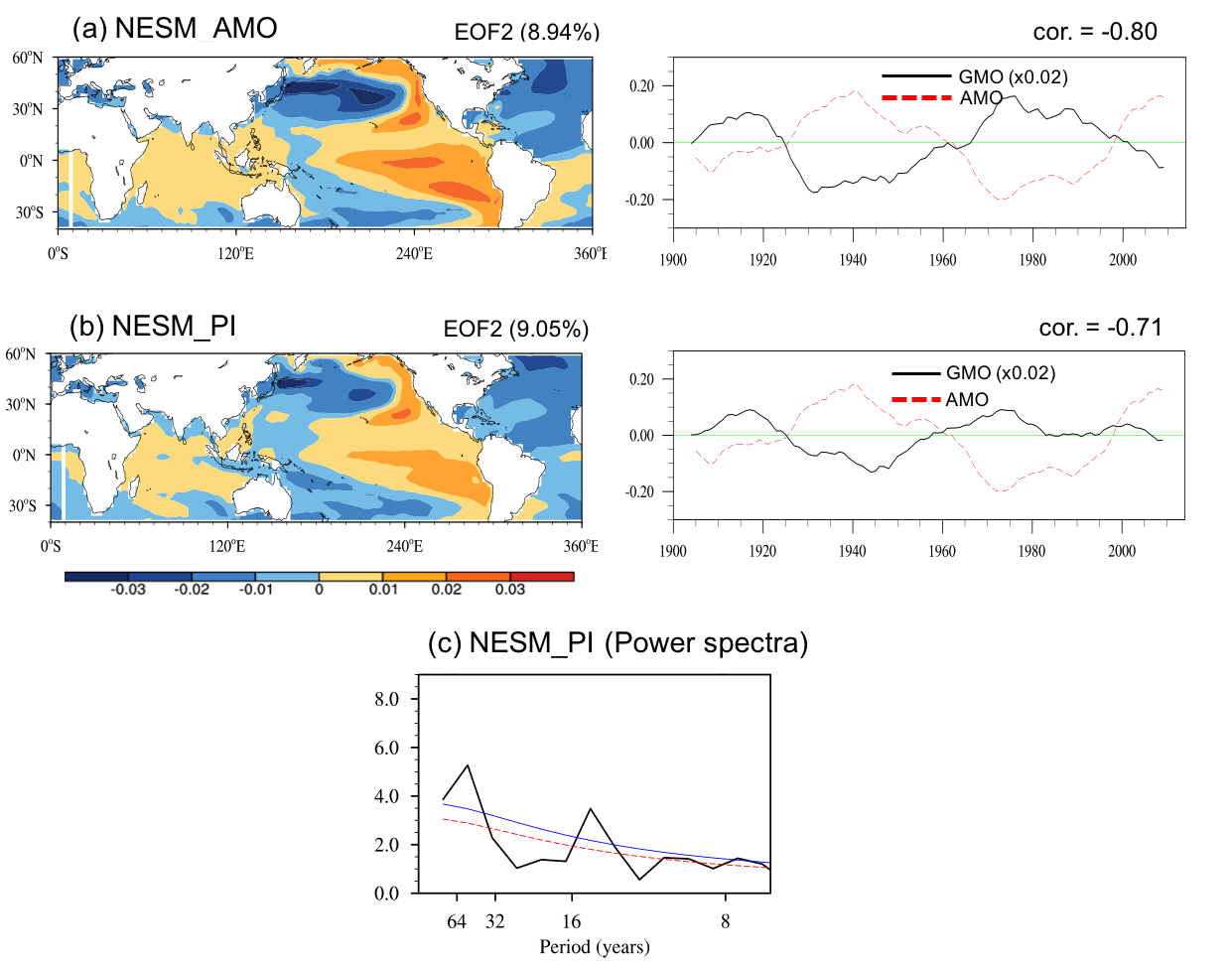
**

**Figure S8.** Horizontal pattern (left panel) and time series of GMV (black line, right panel) and AMO (red line, right panel) from (a) the model with observed SST anomalies over AMO region (0°S–70°N, 80W°–0°W) at decadal timescales (11yrs running mean,1900-2014) and historical external forcings (NESM_AMO) and (b) the model with observed SST anomalies and pre-industrial external forcings (NESM_PI). GMV is defined as the second EOF mode of global SST. AMO is defined as SST averaged over 80W°-0°W, 0°N-70°N). The long-term linear trends in SST data were removed prior to the regression analysis and first and last 11yrs are excluded for analysis.


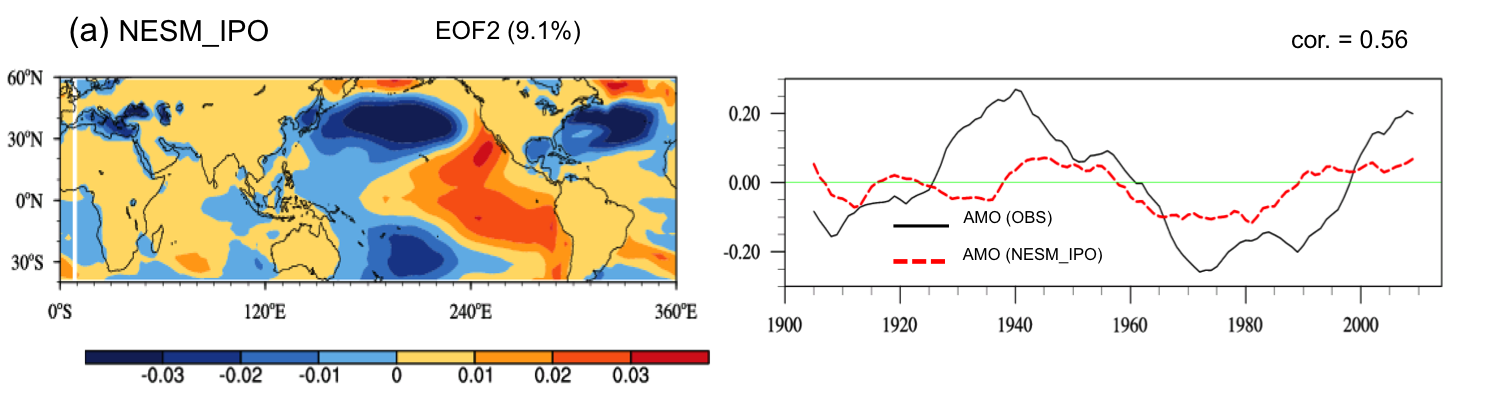


**Figure S9.** Horizontal pattern (left panel) and time series (right panel) of GMV from model simulations with observed SST over the Pacific region (70°S–70°N, 120°–280°E).
